# 

**Published:** 1973-07

**Authors:** 


The
Bristol Medico-Chirurgical Journal
(Incorporating the Medical Journal of the South-West)
Established 1883

Vol. 88 (iii) JULY 1973 No. 327

### BRISTOL MEDICO-CHIRURGICAL JOURNAL

Editor: N. J. Brown, M.B., F.R.C.P., F.R.C.Path.

Published Quarterly

Annual Subscription £2 Post Free

### BRISTOL MEDICO-CHIRURGICAL SOCIETY

**President 1972-73: Dr. James Macrae**

**President-elect: Dr. R. F. Barbour.**

**Honorary Secretary: Dr. I. S. Bailey, 7 Percival Road, Bristol 8.**

**Honorary Treasurer: Dr. Frank Ross, Bristol Royal Infirmary, Bristol 2.**

The Society was founded in 1874. In 1883 publication of the Journal began and has continued quarterly ever since then. During the years 1949 to 1962 it appeared under the title of "The Medical Journal of the South West".

### THE BRISTOL MEDICO-CHIRURGICAL JOURNAL

**Editorial Committee**

| | |
|---|---|
| Editor | Dr. N. J. Brown, Southmead Hospital, Bristol. |
| Assistant Editor | Dr. D. S. Reeves. |
| Members | Dr. Rhys Davies.<br>Dr. M. B. Lennard.<br>Professor R. C. Wofinden.<br>Dr. D. W. Wright.<br>The Hon. Treasurer and Hon. Secretary of the Society. |
| Business Manager | Dr. P. J. F. Baskett, Frenchay Hospital, Bristol. |
| Secretary | Mr. B. P. Jones, The Library, Medical School, University Walk, Bristol BS8 1TD. |

### NOTICE TO CONTRIBUTORS

The Journal is especially concerned to provide a place for the recording of medical thought, interests, and practice in and around Bristol. It therefore welcomes articles that, as well as being of immediate interest to members, will document the local and contemporary medical scene for our successors.

Original articles are invited provided that they have not been published elsewhere. References in the text should be by author's name, with year of publication in parenthesis; they should be listed at the end in alphabetical order, the name of each journal or book being given in full — not abbreviated — and the title of the article added. Illustrations should be supplied ready for reproduction. Line drawing should be in Indian ink on firm white paper or board. The author will be informed if it is found necessary to ask him to pay for the making of blocks.
The following contributions will be especially welcome; notes of forthcoming events, meetings held, degrees conferred, staff appointments, honours and public appointments, and other local news.

### The Medical Library

In 1890 the Bristol Medico-Chirurgical Society founded a medical library which in 1892 was combined with that of the Bristol Medical School. This became the University of Bristol Medical Library in 1925 and an agreement in that year confirmed the privilege of Members of the Society to use the University Medical Library.

### A Selection of Recent Additions to the Medical Library

CIBA FOUNDATION: Personality and science: an interdisciplinary approach. (Churchill Livingstone)

COLQUHOUN, W. P.: Biological rhythm and human performance. (Acad. Press)

DICZFALUSY, E. ed.: Perfusion techniques: transactions of the fourth symposium. (Karolinska Symposia on Research Methods in Reproductive Endocrinology,[4]). (Karolinska Institutet). Presented by Organizing Committee.

DICZFALUSY, E. & BORELL, U. eds.: Control of human fertility (15th Nobel Symposium 1970). (Wiley)

DIETHELM, O.: Medical dissertations of psychiatric interest printed before 1750. (Karger)

ESPIR, L. E. & ROSE, F. C.: Basic neurology of speech. (Blackwell)

FAIRBANKS, V. F. et al.: Clinical disorders of iron metabolism. (Grune & Stratton)

FERRIMAN, D.: Human hair growth in health and disease. (Thomas)

FINCH, S. M. & POZNANSKI, E. O.: Adolescent suicide. (Thomas)

FINLAND, M. et al. eds.: Bacterial infections: changes in their causative agents, trends and possible basis (Bayer Symposium 3). (Springer)

FORD, D. H. ed.: Influence of hormones on the nervous system. (Karger)

GARN, S. M.: Writing the biomedical research paper. (Thomas)

GOOCH, A. et al.: Clues to diagnosis in congenital heart disease (F. A. Davis)

GORTVAY, G. & ZOLTAN, I.: Semmelweiss: his life and work. (Akad. Kiadó)

GREENWALT, T. J. & JAMIESON, G. A. eds.: Formation and destruction of blood cells. (Lippincott/ Blackwell)

GURR, M. I. & JAMES, A. T.: Lipid biochemistry. (Chapman & Hall)

HARDY, J. D.: Human organ support and replacement: transplantation and artificial prostheses. (Thomas)

HARRIS, F.: Paediatric fluid therapy. (Blackwell)

HARRIS, P. & OPIE, L. eds.: Calcium and the heart. (Acad. Press)

HARTLAND, J. & TINKLER, S.: Medical and dental hypnosis and its clinical application. Ed. 2. (Baillière)

HO, B. T. & McISAAC, W. M. eds.: Brain chemistry and mental disease. (Plenum Press)

HOTCHIN, J.: Persistent and slow virus infections (Monographs in virology, 3). (Karger)

INSTITUTION OF ELECTRICAL ENGINEERS: Computers for analysis and control in medical and biological research. (I.E.E.)

JACOBSON, M. A.: Developmental neurobiology. (Holt)

JACOBSON, S. A.: The comparative pathology of the tumours of bone. (Thomas)

JAMES, C. C. M. et al.: Spinal dysraphism. (Butterworth)

KAPANDJI, I. A.: The physiology of the joints. Ed. 2. 2 vols. (Livingstone)

PAPPER, S.: Clinical nephrology. (Little, Brown)

PARK, W. W.: Choriocarcinoma: a study of its pathology. (Heinemann)

PARKER, F. S.: Applications of infra red spectroscopy in biochemistry, biology and medicine. (Hilger)

PERLOFF, J. K.: The clinical recognition of congenital heart disease. (Saunders)

PORCELLATI, G. & DI JESO, F.: Membrane-bound enzymes. (Plenum Press)

PRICE, C. C.: Geometry of molecules. (McGraw-Hill)

PURCHASE, I. F. H. ed.: Mycotoxins in human health. (Macmillan)

RETTERSTOL, N.: Long-term prognosis after attempted suicide. (Universitetsforlaget)

RODDIE, I. C.: Physiology for practitioners. (Churchill Livingstone)

RUBENSTEIN, E.: Intensive medical care. (McGraw-Hill)

RUSCAK, M. & RUSCAKOVA, D.: Metabolism of the nerve tissue in relation to ion movements in vitro and in situ. (University Park Press)

RUSINOV, V. S. ed.: Electrophysiology of the central nervous system. (Plenum Press)

SCHULTZ, D. R.: The complement system (Karger)

SINGLETON, E. B. & WAGNER, M. L.: Radiologic atlas of pulmonary abnormalities in children. (Saunders)

SLATER, E. & COWIE, V.: The genetics of mental disorders. (Oxford Univ. Press)

SPELLER, S. R.: Law relating to hospitals and kindred institutions. Ed. 5. (Lewis)

SWAIMAN, K. F. & WRIGHT, F. S.: Neuromuscular diseases of infancy and childhood. (Thomas)

TRAISMAN, H. S.: Management of juvenile diabetes mellitus. Ed. 2. (Mosby/Kimpton)

TRUETA, J.: Gathorne Robert Girdlestone. (Oxford Univ. Pres for Girdlestone Orthopaedic Society)

TRUMPY, J. H.: Transneuronal degeneration in the pontine nuclei of the cat. (Springer)

WALDVOGEL, F. A. et al.: Osteomyelitis. (Thomas)

WILSON, R. N.: The sociology of health: an introduction. (Random)

ZILVA, J. F. & PANNALL, P. R.: Clinical chemistry in diagnosis and treatment. (Lloyd-Luke)

### Notes and News

**F.R.C.P.**
D. Burman
A. C. Fairburn
J. B. Gordon-Russell
L. Haas
J. Verrier Jones
J. Russell Rees
H. Urich

**F.R.C.O.G.**
Audrey Midwinter

**F.R.C.Path.**
B. B. Beeson
M. S. Dunnill
Joan Guy
B. H. Ruebner
L. A. D. Tovey

**M.R.C.P.**
M. R. Wills

Dr. J. Jancar, Consultant Psychiatrist, Stoke Park Hospital, will deliver the Blake Marsh Lecture at the Royal College of Psychiatrists on 4th February, 1974. His subject will be "Neuro-cutaneous Disorders and Mental Functioning".

Professor M. A. Epstein has been awarded the 1973 Paul-Ehrlich and Ludwig-Darmstaedter Prize and Medal of the West German Paul Ehrlich Foundation.

Dr. J. F. Skone has been invited to be Visiting Professor in Community Health in the University of Auckland and has also been appointed to one of the Secretary of State's short-term fellowships to act as tutor in the first multi-professional course in the management of integrated health care in the University of Manchester in April-May, 1972.

Dr. E. Rhys Davies has been elected Honorary Secretary of the British Nuclear Medicine Society.

Dr. Sarah Walker retired from her position of Principal Medical Officer (Maternal and Child Health) for the City of Bristol on 21st June, 1973, after more than twenty years' distinguished service. Consequent upon her retirement a new post has been established of Principal Medical Officer (Child Health) which will be filled by Dr. Marie Freeman, formerly a Medical Officer in Merton and previously School Medical Officer at High Wycombe.

Dr. John Apley delivered the Charles West Lecture at the Royal College of Physicians, London on 7th February, 1973. His subject was, " 'Which of you by taking thought can add one cubit unto his stature?' Psychosomatic illness in children: A Modern Synthesis".

### Notice

### British Association of Surgical Oncology

The first Annual General Meeting, Scientific Meeting and Dinner of the Association was held at the Royal College of Surgeons of England on Friday, 8th June when the subject of the Scientific Meeting was "Should lymphadenectomy be discarded?"

Membership of the Society shall be surgeons elected because of their interest in Oncology. Associate Members shall be elected from among other specialists in Oncology who are engaged in related disciplines such as radiotherapy, chemotherapy, immunotherapy, etc.

The annual membership subscription is £8.50. Membership is limited. Full details can be obtained from The Secretary of the Association, Mr. Ian Burn, F.R.C.S., Hon. Secretary, British Association of Surgical Oncology, Royal College of Surgeons of England, Lincolns Inn Fields, London WC2A 3PN.

The National Committee invites applications for Membership from all who wish to become Founder members of the Association and applications should be sent by letter to the Honorary Secretary.

### University of Bristol

### Examinations Results and Dissertations Approved

**M.D.**
David John GOLDIE
George SAKALIS

**Ch.M.**
Michael O'DRISCOLL

**Ph.D.** (Faculty of Medicine)
Lydden Robert POLLEY
John Andre SMITH
Thangarajah SOORIYAMOORTHY

**D.P.H.**
Örn BJARNASON
Robert CHAPPLE
Jose Celestino CHACIN
John Court CORNWELL
Neelambal DURAISWAMY
Morag Lillie INSLEY
Nils Knud ROSDAHL
Hasteinn Brynleifur STEINGRIMSSON
Ranjit Kumar SENGUPTA
Freda VERGHESE
Kenneth William WOOLHEAD

**M.B. Ch.B.**
**WITH HONOURS**
Colette BUTLER—with Distinction in Child Health
Christine Gillian COUSINS — with Distinction in Public Health
Helen Jane HARRIES—with Distinctions in Public Health and in Child Health
Lyn Margaret JACKSON — with Distinctions in Obstetrics and Gynaecology, Mental Health and in Child Health
Craig Stanley MELLOR—with Distinctions in Surgery, Mental Health and in Child Health.
Moira Ann PINKNEY—with Distinctions in Clinical Pathology and in Public Health.

**PASS**
Abdulhamid Suleman ABDULREHMAN
Elizabeth Jane ANSTEY
Jana Susanne Helena BAILEY
Elizabeth Ann BALMER
David BAREFORD
Henry Louise Robert BASTIAENEN
Ashni BEHAL
Grant Frederick Allen BENFIELD
Almass Gulamhussein BHANJI
Moira Ann BINGHAM
Ingrid Margaret BOWDLER
David Edward Robertson BURT
Ilona Wanda CHANTLER
Colin Michael CHAPMAN
John Edwin Henry CHAPMAN
Stephen George CLARKSON
Peter Campbell CLIFFORD
Michael Joheph COOKE
Vincent COOPER
Peter Francis COXHEAD
Richard Alan DAVIES
Parvin DHALLA
Jacqueline Claire DICKINS
Malcolm David Cairns DONALDSON
John William EASTGATE
Howard Owen FAIRCLOTH
Jennifer Ann FENTON
Huw Barie James FISCHER
Timothy James FITZSIMONS
Christopher James FOSTER
Richard Michael GANT
Duncan Stuart GOODLAND — with Distinction in Public Health
Simon Michael Wyatt GRAINGE
Philip Anthony GREEN
Liam Patrick GRIFFIN
Philip Andrew HALL
Leila Beatrice HARRIS
Nadine HARRISON
Peter Alexander HASSALL
John Philip HEATHCOCK
Andrew Peter HICKS
Raymond John HILL
Rury Reginald HOLMAN
Paul William Jones HOUGHTON
Martin Lloyd HOWELL
Christopher Courtenay HULBERT—with Distinction in Public Health
David John Robert JEFFRIES
Roy Malcolm JONES
Keith Charles JUDKINS
Alan John KERRIGAN
Stephen Ralph KETTLE
Judith Eve KINGSTON—with Distinction in Public Health
Peter Stanley KLIMIUK
Bernard Von KUENSSBERG
Jonathan Ellis LADD
Rowena Anne LASS
Alfred Edward LEDNER
Philip John LEIBERMAN
Erle Rodney LITTLEWOOD
Christopher Roland LOVELL
Robert Henry MARTIN
Andrew Henry MASLOWSKI
Wendy Jane MASON
Nicholas Stephen MILLIGAN

David Malcolm MILNE
Shiu Kow MING
Yusufali Akberali MNYUSIWALLA
Susan Carol MURPHY
Peter Melville O'HARE
John OSMAN—with Distinction in Clinical Pathology
Diana Rosemary PADFIELD
David Elliott PELTA
Richard Duncan POCOCK
Philip Hugh POWELL
Michael Patrick RAFFERTY
Philip Edward RANDALL
Farial Fatehali Dharmasi RAWJI
Andrew John RAWLINSON
David Henry READ
Alison Patricia ROBERTS
David Gordon Islay SCOTT
Rosemary Anne SILK
Philip Arthur SIMMONS
Elizabeth Romana SINCLAIR
Stephen Ian STEINHARDT
Stephen Charles Mayne SWEET
John Daniel MacPherson TAYLOR
Catriona Macbeth THOMSON
Paul Alexander TOMLINSON
Philip John TOPLIS
Keith WAKELIN
Zainab Rahemtulla WALJI
Ruth Marilyn WARWICK
David Bryant WHELDON
Martin Highfield WISE
Margaret Clair YHAP

